# Impact of pediatric cancer on family relationships

**DOI:** 10.1002/cam4.1393

**Published:** 2018-03-25

**Authors:** Craig Erker, Ke Yan, Liyun Zhang, Kristin Bingen, Kathryn E. Flynn, Julie Panepinto

**Affiliations:** ^1^ Department of Pediatrics Cincinnati Children's Hospital Medical Center Cincinnati Ohio; ^2^ Department of Pediatrics Medical College of Wisconsin Milwaukee Wisconsin; ^3^ Department of Medicine Medical College of Wisconsin Milwaukee Wisconsin

**Keywords:** Family relationships, pediatric oncology, PROMIS, quality of life, siblings

## Abstract

Little is known about the impact of cancer on family relationships from the perspective of the pediatric cancer patient and their sibling(s). This study assessed and compared children's experiences of family relationships in patients receiving active cancer therapy, those who have completed therapy, and siblings. A cross‐sectional study of children with cancer and their siblings aged 8–17 years old was conducted. Children completed the PROMIS Pediatric Family Relationships short form and the Depressive Symptoms, Anxiety, and Peer Relationships short forms. The Mann–Whitney test assessed differences in Family Relationships scores between therapy groups, while the Wilcoxon signed‐rank test assessed differences between patients and siblings. An actor–partner interdependence model (APIM) was used to assess how patient and sibling variables were associated with their own and each others’ family relationships. Two hundred and sixty‐five children completed the assessments. Siblings of patients on‐therapy had worse family relationships than patients on‐therapy (*P* = 0.015). Family relationships of patients off‐therapy did not differ from their siblings or the patients on‐therapy. Family relationships scores did not differ between the sibling cohorts. The APIM found patient family relationships were impaired when their own peer relationships decreased and when either their own or their siblings had increased depressive symptoms. Sibling family relationships were impaired when their own depression increased, and when the patient counterpart was female, younger age, had less depressive symptoms, more anxiety or a diagnosis of leukemia/lymphoma (compared to solid tumor). Based on these findings, increased psychosocial resources for patients and siblings of children undergoing cancer therapy may be warranted.

## Introduction

Children with cancer can experience decreased physical, emotional, and social health‐related quality of life (HRQoL) compared to healthy children 1. Poor family functioning in children with cancer has been shown to negatively influence a child's HRQoL and impair their ability to properly adjust 2, 3. This supports the critical role of the family for children impacted by cancer.

Children receiving active cancer therapy and cancer survivors experience increased impairments in behavioral and social domains compared to controls 4, 5. Although many families adjust well to pediatric cancer 6, some families may develop sustained poor functioning 3, 7. However, longitudinal studies of pediatric cancer patients suggest that overall most HRQoL domains improve overtime, including social health 5, 6, 8, 9, 10.

During cancer treatment, siblings are overlooked family members, and up to 63% can have adjustment difficulties 11. A meta‐analysis showed that siblings of children with cancer and other chronic illnesses experience more depression, anxiety, and worse peer relationships than siblings without a chronically ill brother or sister 12. During a patient's treatment for cancer, some siblings report feeling lonely and report decreased attention 13. Siblings may cognitively understand their brother or sister's illness and increased needs but can still exhibit impaired social and emotional HRQoL 14. Even 2 years after a child completes cancer treatment, a sibling's emotional and social problems can continue 15.

Family relationships can be influenced by many variables. Depression, anxiety, and a child's peer relationships have previously been associated with family relationships in both oncology and nononcology patients 16, 17, 18. Socioeconomic status can also influence a child's well‐being and their relationships 19, 20.

In this study, we assessed the subjective experiences of family relationships in children with cancer and their siblings using the Patient‐Reported Outcomes Measurement Information System^®^ (PROMIS^®^) Pediatric Family Relationships measure. We hypothesized that siblings would have more impairment in family relationships compared to their brother or sister with cancer and that children receiving cancer therapy would report more impairment in family relationships than children who completed cancer therapy. We also explored factors that may be associated with family relationships in children with cancer and their siblings.

## Methods

### Study design and study population

A cross‐sectional study was conducted using a convenience sample of oncology patients and their siblings, aged 8–17 years old, at a single institution from October 2015 to December 2016. The Children's Hospital of Wisconsin is a large tertiary care facility located in Milwaukee, Wisconsin that cares for the majority of children in southeast Wisconsin. Children were recruited into one of four cohorts based on inclusion and exclusion criteria. Parallel data were also collected from caregivers for each subject. The study was approved by the Institutional Review Board prior to enrollment.

Cohort 1 involved patients who were currently receiving cancer therapy (patients on‐therapy). Inclusion criteria included the diagnosis of an oncologic process, currently receiving chemotherapy and/or radiation therapy, and greater than 4 weeks into treatment. Cohort 2 involved patients who had completed cancer therapy (patients off‐therapy). Inclusion criteria included the diagnosis of an oncologic process that required chemotherapy and/or radiation therapy and completed their cancer treatment more than 6 months ago. Cohorts 3 and 4 involved siblings of participants in cohorts 1 and 2 (siblings of patients on‐therapy and siblings of patients off‐therapy). Siblings had to live with the same caregivers as the patient at least 50% of the time and no more than two siblings per family could enroll. All four cohorts had the same exclusion criteria of (1) children with severe cognitive impairment as determined by the clinical team; (2) non‐English literate. Patients who received surgery alone for a tumor were considered to have least intensive therapy and were not included 21.

### Study procedure

Identification of eligible patients on‐ and off‐therapy was completed weekly using the electronic medical record. Patients were screened using inclusion and exclusion criteria. Eligible patients were then approached by a member of the research team in the oncology clinic or inpatient unit during scheduled clinic visits or inpatient stays. At the time of enrollment, caregivers were asked if the patient's siblings would consider participation. With caregiver permission, siblings were approached to consent for the study.

PROMIS Assessment Center was used to collect all data. Data were collected from patients and caregivers in person via electronic tablets. Siblings of patients not available for in‐person completion were contacted and assented by phone. Study personnel emailed assessment links for the siblings to complete online. If families did not have Internet access, the siblings had questions read to them over the phone. Previously, different modes of measure administration resulted in comparable scores 22. Siblings were contacted up to three times to complete the assessments. Parents were instructed not to assist patients or siblings with their assessments, regardless of age. All children were expected to complete the assessments on their own. If a child needed the questions read to them, due to impaired vision or inability to access email, clarifications were discouraged. If a child felt like they were not able to understand or answer a question, they were instructed to skip the question. We attempted to collect patient and sibling assessments within 7 days of each other, but data were not excluded if this time frame was not met.

### Measures and variables

#### Primary outcome

PROMIS Family Relationships *T*‐score. Current family assessment tools commonly evaluate the family as a whole, consist of numerous questions, and rely on parent report. The limited number of existing child‐report measures is often validated for older children and do not assess subjective family experiences 3, 19, 20, 23, 24. The PROMIS^®^ Pediatric Family Relationships measure was developed and validated to addresses these gaps 25. The PROMIS Family Relationships measure was informed by theories of attachment, bioecological influences on health and living systems which suggest that relationships and illness dynamically affect one another 25. The Family Relationships domain was developed following NIH PROMIS standards using a rigorous mixed‐method instrument development process 26. PROMIS was developed for use in both healthy populations and those with medical conditions 27, 28. Consistent with other pediatric PROMIS domains, the Family Relationships self‐report instrument was validated for children aged 8–17 years old along with a parallel parent report for children aged 5–17 years. The 8‐item short form was used for both child self‐report and parent report. The items use a 5‐point response scale (never to always) and have a 4‐week recall period. A mean score of 50 (standard deviation [SD] of 10) corresponds to the US average. The *T*‐score was calculated using item response theory parameters which were established during measure development 25. Lower scores indicate worse family relationships.

#### Covariates of interest

Several other variables were assessed for their association with family relationships. These included emotional and social health measures using child self‐report of PROMIS Depressive Symptoms short form 4b, PROMIS Anxiety short form 4b, and PROMIS Peer Relationships short form 4a. These domains each consisted of 4‐items for the child self‐report while the parent‐proxy‐report used 8‐item short forms. The items use a 5‐point response scale (never to always) and have a 7‐day recall period. Higher scores on the Depressive Symptoms and Anxiety measures and lower scores on the Peer Relationships measure indicate domain impairment.

Other covariates assessed were demographic and diagnostic information collected from caregivers including patient's cancer diagnosis category, subject's age, study group (on‐therapy, off‐therapy or sibling), and level of socioeconomic deprivation. The area deprivation index (ADI) is used as a surrogate for socioeconomic status 29.

### Statistical analysis

Descriptive statistics were summarized for demographic information and clinical data. A contingency table with chi‐squared test was used to examine the relationship between categorical variables. For contingency tables that had more than 20% of cells with an expected value of less than 5, a Fisher's exact test was used. The Mann–Whitney test was used to compare the child's age for nonmatched samples, and the Wilcoxon signed‐rank test was completed for matched samples.

This study aimed to enroll 64 subjects from each group in order to detect a difference in *T*‐scores of 5 or more using an alpha of 0.05 and a power of 0.80. Intergroup comparisons were then assessed. Again, the Mann–Whitney test was utilized for nonmatched samples, while the Wilcoxon signed‐rank test was used for matched samples. As siblings within the same family could not be analyzed in an independent manner, only the sibling closest in age to the patient was utilized in the matched analysis and subsequent actor–partner interdependence model (APIM) analysis. The internal consistency reliability of the Family Relationships measure was assessed using Cronbach's alpha statistic for both patients and siblings.

To assess which covariates of interest predict better or worse family relationships, an APIM was used to analyze data from patients and siblings together as a dyad 30. The maximum likelihood method was used to estimate the covariance parameters. A compound symmetry covariance structure was used to assign an equal amount of nonindependence to dyad members. The following variables were considered in the APIM assessments: gender, age, peer relationships, depressive symptoms, and anxiety from both actor and partner sides. Also, on versus off‐therapy, diagnosis category and ADI were assessed and were included as the same variable for patients and siblings in the model. This model allowed for assessment of one variable while controlling for others. Depending on how a variable influenced patient and sibling family relationships, a pattern was established. A pattern could be classified as actor‐only, partner‐only, couple (equal actor and partner effects), or contrast (equal actor and partner effects, but opposite signs) using estimate ratios 31. A *k* value, which is the ratio of the partner estimate to the actor estimate, was used to assess these patterns. A *k *=* *1 demonstrates a perfect couple relationships, whereas a *k *=* *−1 indicates a perfect contrast relationship. For final models, a *P‐*value of less than 0.05 was considered significant. SAS 9.4 (SAS Institute, Cary, NC) was used to perform statistical analyses. The same analyses were completed for parent reports in parallel with child self‐report data.

## Results

### Participant completion, demographics, reliability

**Figure 1:**
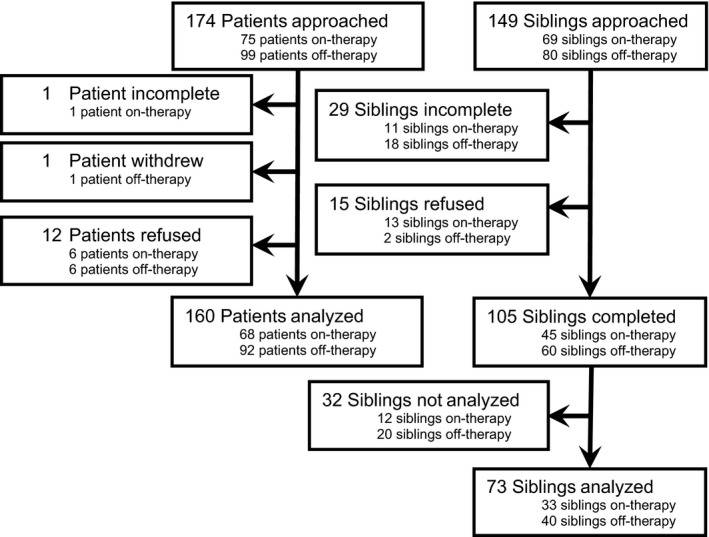
Study enrollment flowchart.

One hundred and seventy‐four patients and 149 siblings were approached. Sixty‐eight patients on‐therapy and 92 patients off‐therapy completed the study along with 45 siblings of patients on‐therapy and 60 siblings of patients off‐therapy (Figure 1). Incomplete assessments occurred when a subject was consented by their guardian, but the subject decided not to complete the assessment. The majority of incomplete assessments for siblings occurred because of inability to contact the subject by phone or email. Study refusal decisions were not explored. The time to complete the questionnaire was 10–15 min, and the median interval for patient and siblings to complete the Family Relationships measure was 2 days (0–27 days).

There were no statistically significant differences in child age, gender, race, ethnicity, parental educational level, parental marital status, number of adults in the household, number of people in the home, diagnosis groups, treatment type, and relapse percentage between patients on‐ and off‐therapy (*P* > 0.05) or between siblings of patients on‐ or off‐therapy (*P* > 0.05, Table 1). Also, there was acceptable internal reliability of the 8‐item Family Relationships measure for both patients and siblings with Cronbach's alpha statistics of 0.835 and 0.885, respectively.

**Table 1:** Demographic characteristics of child participants by study cohort

	Patients on‐therapy *n* = 68	Patients off‐therapy *n* = 92	Siblings on‐therapy *n* = 33	Siblings off‐therapy *n* = 40
Age in years (median, range)	12 (8–17)	13 (8–17)	13 (8–17)	13 (8–17)
Gender (*N*, %)				
Male	33 (49)	52 (57)	14 (42)	21 (53)
Race (*N*, %)				
White	53 (79)	75 (82)	26 (79)	31 (78)
Black	5 (8)	6 (7)	4 (12)	2 (5)
Other	9 (13)	10 (11)	3 (9)	7 (17)
Ethnicity (*N*, %)				
Hispanic/Latino	5 (7)	4 (4)	2 (6)	3 (8)
Not Hispanic/Latino	61 (90)	87 (95)	30 (94)	37 (93)
Not reported	2 (3)	1 (1)	1 (3)	0
Parental educational attainment (*N*, %)				
High school or less	16 (24)	15 (16)	7 (21)	6 (15)
Some college (no degree)	17 (25)	29 (32)	5 (15)	9 (23)
Associate or bachelor degree	24 (35)	33 (36)	13 (39)	15 (38)
Advanced or professional degree	11 (16)	15 (16)	8 (24)	10 (25)
Adults (>18 years) in household (*N*, %)				
Single adult household	10 (15)	15 (16)	5 (15)	6 (15)
Two or more adult household	58 (85)	77 (84)	28 (85)	34 (85)
Marital status (*N*, %)				
Married or living with partner	48 (71)	70 (77)	25 (76)	32 (80)
Divorced/separated	11 (16)	14 (15)	2 (6)	4 (10)
Never married/other	9 (13)	7 (8)	6 (18)	4 (10)
Number of people in the home (median, range)	4 (1–10)	4 (2–9)	4 (3–10)	4 (3–9)
Diagnosis group of patient (*N*, %)				
Leukemia/lymphoma	41 (60)	57 (62)	22 (67)	27 (68)
Solid tumor	17 (25)	22 (24)	4 (12)	9 (22)
CNS tumor	10 (15)	13 (14)	7 (21)	4 (10)
Treatment type (*N*, %)				
Chemotherapy only	42 (63)	46 (52)	N/A	N/A
Radiation only	0 (0)	3 (2)		
Combination^a^	23 (34)	33 (37)		
BMT	2 (3)	7 (8)		
Relapse (*N*, %)				
Yes	16 (24)	11 (12)	N/A	N/A

a Combination of chemotherapy and/or radiation +/− surgery.

### Family relationships comparisons for matched subjects

Child self‐report comparisons between patient and sibling groups show that siblings on‐therapy had worse family relationships scores than their brothers or sisters on‐therapy (*P* = 0.015, Table 2). Comparison of family relationships *T*‐scores between patients off‐therapy and siblings of patients off‐therapy showed no difference (*P* = 0.082).

**Table 2:** Family relationships *T*‐score comparisons of matched subjects

Cohort	*N*	Patient median *T*‐score (IQR)	Siblings median *T*‐score (IQR)	Median (IQR) difference	*P*‐value
Child self‐report					
On‐therapy	33	47.1 (43.0, 51.6)	45.5 (37.4, 49.5)	3.2 (−1.4, 8.5)	0.015
Off‐therapy	40	47.5 (42.6, 52.4)	46.8 (42.3, 51.6)	4.3 (−4.1, 8.3)	0.082
Parent report					
On‐therapy	33	49.4 (43.7, 57.6)	42.1 (36.7, 46.1)	6.4 (2.9, 12.0)	<0.0001
Off‐therapy	40	47.7 (42.1, 53.0)	45.0 (38.1, 54.4)	0.3 (−1.3, 6.8)	0.16

Consistent with child self‐report data but larger in magnitude, family relationships reported by parents show siblings of patients on‐therapy have worse family relationships scores than patients on‐therapy (*P* < 0.0001). There was no significant difference when comparing parent reports of patients off‐therapy to siblings of patients off‐therapy (*P* = 0.16).

### Family relationships comparisons for nonmatched cohorts

There was no difference in children's experiences of family relationships between patients on‐therapy and patients off‐therapy (*P* = 0.44, Table 3). Likewise, comparison of scores between siblings of patients on‐therapy and siblings of patients off‐therapy showed no significant difference (*P* = 0.22).

**Table 3:** Family relationships *T*‐score comparisons of nonmatched subjects

Cohort	On‐therapy		Off‐therapy		*P*‐value
	*N*	Median (IQR)	*N*	Median (IQR)	
Child self‐report					
Patients	68	48.7 (44.6, 52.6)	92	49.0 (43.8, 55.8)	0.44
Siblings	33	45.5 (37.4, 49.5)	40	46.8 (42.3, 51.6)	0.22
Parent report					
Patients	68	49.4 (43.1, 57.6)	92	48.5 (42.6, 55.5)	0.65
Siblings	33	42.1 (36.7, 46.1)	40	45.0 (38.1, 54.4)	0.21

Consistent with child self‐report data, family relationships reported by parents of patients on‐therapy and patients off‐therapy (*P* *=* 0.65) as well as of siblings of patients on‐therapy and siblings of patients off‐therapy showed no difference (*P* = 0.21).

### Actor–partner interdependence model for child self‐report

Seventy‐three pairs of patient sibling dyads were available for assessment using APIM. This model showed that siblings of patients with solid tumors were found to have better family relationships than siblings of patients with leukemia/lymphoma. Therapy group (on‐ or off‐therapy) was not significantly associated with family relationships for patients or siblings. In addition, ADI level was not significant.

Variables that had significant actor or partner effects and led to impairment in the patients’ family relationships scores were as follows: (1) worse patient peer relationships, (2) higher patient depression, and (3) higher sibling depression. Variables that had significant actor or partner effects and led to impaired sibling family relationships scores were as follows: (1) patient being of female gender, (2) lower patient age, (3) lower patient depression, (4) higher patient anxiety, and (5) higher sibling depression (Table 4). Depressive symptom scores had both an actor and a partner effect on patient and sibling family relationships. Depressive symptoms demonstrated a couple pattern on patient family relationships (*k *=* *0.81) where increasing patient and sibling depressive scores were negatively associated with family relationships. In sibling family relationships, depressive symptoms showed a contrast pattern (*k *=* *−0.83) where family relationships decreased when their own depressive symptoms increased but improved when patients experienced more depressive symptoms.

**Table 4:** APIM analysis of patient and sibling dyad family relationships

Variable	Actor or partner effect	Estimate (SE)	*P*‐value
Patient solid tumor diagnosis	Partner	5.43 (1.87)	0.0004
Patient male gender	Partner	3.41 (1.52)	0.0258
Patient age	Partner	0.61 (0.29)	0.0328
Patient depression	Partner	0.40 (0.14)	0.0047
Patient anxiety	Partner	−0.33 (0.11)	0.0048
Sibling depression	Partner	−0.31 (0.10)	0.0031
Sibling depression	Actor	−0.48 (0.10)	<0.0001
Patient depression	Actor	−0.38 (0.14)	0.0085
Patient peer relationships	Actor	0.24 (0.08)	0.0051

### Actor–partner interdependence model for parent‐proxy‐reports

Seventy‐three pairs of parent‐proxy data of patient and sibling dyads were available for assessment using APIM. The assessment showed that there was no significant effect on family relationships by therapy groups (on‐ or off‐therapy), diagnoses category, ADI, gender or age. A patients’ family relationships were worse if the sibling had impaired peer relationships. A siblings’ family relationships were worse if the sibling had more depression or worse peer relationships. A siblings’ family relationships were better if the patient had more depression or less anxiet (Table 5). Depressive symptoms had two effects on sibling family relationships and showed a contrast pattern (*k *=* *−0.94) where family relationships decreased when their own depressive symptoms increased but improved when patients experienced more depressive symptoms.

**Table 5:** APIM analysis of parent‐proxy patient and sibling dyad family relationships

Variable	Actor or partner effect	Estimate (SE)	*P*‐value
Sibling peer relationships	Partner	0.27 (0.12)	0.0292
Patient depression	Partner	0.52 (0.20)	0.0132
Patient anxiety	Partner	−0.48 (0.18)	0.0089
Sibling peer relationships	Actor	0.29 (0.12)	0.0202
Sibling depression	Actor	−0.55 (0.18)	0.0025

## Discussion

This study directly compares children with cancer to their siblings using an assessment tool that measures the children's own experience of their family relationships. It was found that siblings of patients on‐therapy have worse family relationship scores than their ill brother or sister, and when off‐therapy, no differences were detected. In addition, we found significant associations between multiple patient and sibling variables on family relationships using a model able to examine patients and siblings as dyads.

The APIM demonstrated multiple factors are associated with how patients and siblings experience family relationships. Patient family relationships worsened as their own peer relationships declined as well as when either their own or their siblings’ depressive symptoms increased. On the other hand, sibling family relationships were largely affected by partner (patient) effects and were worse if the patient had any of the following characteristics: a diagnosis of leukemia/lymphoma, female in gender, younger in age, more anxiety symptoms, and less depressive symptoms. The only variable of their own that was negatively associated with sibling family relationships was an increase in depressive symptoms.

Our finding that as a patient or sibling's depressive symptoms increase, their family relationships worsen, supports the previous literature 32. The association between family relationships and depression in those affected by childhood cancer has also been shown 33. Interestingly, sibling family relationships scores improved when their brother or sister with cancer experienced more depressive symptoms. This result seems contrary to initial judgment but was corroborated by parent‐proxy data. One could hypothesize that siblings felt the need to be more connected to their family when their brother or sister with cancer was struggling. On the other hand, a patient may be more hopeless if their healthy sibling is also struggling with depressive symptoms. A similar relationship was previously described in married couples, showing depressive symptoms in a partner can negatively impact a patient's quality of life 34. Patient anxiety was found to be negatively associated with sibling family relationships in both the child and proxy‐reports. It is well known that anxiety and depression are frequently comorbid conditions 35. Clinicians should consider both depressive symptoms and anxiety when assessing family relationships.

For patients, better peer relationships were associated with better family relationships. This highlights the need for strong peer support in children with cancer 36, and demonstrates the importance of psychosocial interventions to optimize the positive effect of their social support system. Only the parent‐proxy‐report, and not the sibling self‐report, showed a positive association between sibling peer relationships and improved family relationships. Peer Relationships scores have previously been documented as having higher item‐level discrepancy between proxy and child reports possibly due to its difficult to observe nature 37. However, the previous literature has shown that siblings of patients with cancer report similar peer relationships as compared to their healthy classmates 38.

Siblings of patients with leukemia/lymphoma experienced worse family relationships than children with solid tumors. Also, siblings of female patients, younger patients, and those with more anxiety had worse family relationships. These variables, however, did not impact the patient's own family relationships. A previous study has shown that patients with leukemia/lymphoma overall experience worse HRQoL, especially in the first 6 months of therapy 39. Along with younger age, leukemia/lymphoma diagnosis may require more attention by family members leading a sibling to feel less important and connected to their family. These findings highlight how family relationships of siblings are associated with several patient factors that may or may not be actionable. For example, a patient's anxiety symptoms may be improved through psychology intervention, which may, in turn, improve a sibling's family relationships, while other variables such as the patient's diagnosis, age, and gender are not actionable.

The study was limited by its cross‐sectional design and its inability to measure family relationships change overtime. Also, many pediatric oncology patients did not have eligible siblings, which reduced sibling enrollment. We did not look specifically at how many families relocated or spent time at temporary living facilities, which may affect family relationships by contributing to separation. It is important to recognize that predictors of family relationships may vary depending on which covariates are included and how the analyses are structured. Lastly, it is not yet clear what a meaningful difference in *T*‐score is for the PROMIS Family Relationship domain. Other PROMIS domains have set meaningful differences at 2–3 points 40. Some of the aforementioned limitations can be overcome with prospective studies and determining clinically meaningful scores. At this time, screening tools to identify families that would benefit from increased social resources and family‐directed interventions are limited 41. Further work with the PROMIS Pediatric Family Relationships measure could be pursued as a screening tool to target families and individuals that would most benefit from specific interventions 42.

## Conclusion

The PROMIS Family Relationships measure is a reliable measure for the pediatric oncology population. Siblings of patients receiving therapy for cancer report worse family relationships than their ill brother or sister. A patient's family relationships are associated with their own level peer relationships and both their own and their siblings’ level of depressive symptoms. Sibling family relationships scores are associated with their own level of depression and multiple factors from their brother or sister with a cancer diagnosis. Increased psychosocial resources for families of children undergoing cancer therapy, particularly siblings, may be warranted. Incorporation of the PROMIS Pediatric Family Relationships measure into clinical practice may help shape social awareness of the oncology population and identify children that could benefit from increased family support.

## Conflict of Interest

None declared.
